# Molecular Motor KIF3B Acts as a Key Regulator of Dendritic Architecture in Cortical Neurons

**DOI:** 10.3389/fncel.2020.521199

**Published:** 2020-10-19

**Authors:** Nadine F. Joseph, Eddie Grinman, Supriya Swarnkar, Sathyanarayanan V. Puthanveettil

**Affiliations:** ^1^The Skaggs Graduate School of Chemical and Biological Sciences, The Scripps Research Institute, La Jolla, CA, United States; ^2^Department of Neuroscience, The Scripps Research Institute, Jupiter, FL, United States

**Keywords:** kinesins, KIF3B, dendritic arborization, spine density, spine morphology, structural plasticity, mitotic KIF

## Abstract

Neurons require a well-coordinated intercellular transport system to maintain their normal cellular function and morphology. The kinesin family of proteins (KIFs) fills this role by regulating the transport of a diverse array of cargos in post-mitotic cells. On the other hand, in mitotic cells, KIFs facilitate the fidelity of the cellular division machinery. Though certain mitotic KIFs function in post-mitotic neurons, little is known about them. We studied the role of a mitotic KIF (KIF3B) in neuronal architecture. We find that the RNAi mediated knockdown of KIF3B in primary cortical neurons resulted in an increase in spine density; the number of thin and mushroom spines; and dendritic branching. Consistent with the change in spine density, we observed a specific increase in the distribution of the excitatory post-synaptic protein, PSD-95 in KIF3B knockdown neurons. Interestingly, overexpression of KIF3B produced a reduction in spine density, in particular mushroom spines, and a decrease in dendritic branching. These studies suggest that KIF3B is a key determinant of cortical neuron morphology and that it functions as an inhibitory constraint on structural plasticity, further illuminating the significance of mitotic KIFs in post-mitotic neurons.

## Introduction

Neurons are senescent, multipolar cells that require specialized transport machinery to maintain normal cellular function, and morphology. Molecular motor proteins known as the kinesin family of proteins (KIFs) are vital players in this intracellular transport system in post-mitotic neurons. KIFs use ATP hydrolysis to generate force to bind and move along the microtubule (MT) cytoskeleton to transport cargos of RNA, organelles, and proteins to distal areas of the cell (Hirokawa, 1998; Puthanveettil et al., 2008; Puthanveettil, 2013). KIFs form a large class of proteins, with phylogenic analysis identifying 14 different families with 45 members (Miki et al., 2001). KIF function can be separated into two broad groups based on the cell types in which it is localized: modulation of the cytoskeleton in mitotic cells and trafficking of cargos in post-mitotic cells.

KIFs have highly specialized roles in developing cells. Several KIFs have been shown to facilitate cell division by aiding the movement of machinery required for cytokinesis as well as transporting mitotic specific cargos. KIF4 aids in the formation of the spindle fibers (Kurasawa et al., 2004), while KIF11 is involved in the fidelity of chromosomal segregation by ensuring the structural integrity as well as the movement of the spindle fibers (He et al., 2016). These mitotic KIFs have recently been shown to have a post-mitotic role. Work described in Swarnkar et al. (2018) described that KIF11 inhibits synaptic transmission in a pre-synaptic manner *via* the regulation of the presynaptic protein, piccolo.

Loss of function, genetic, and biochemical studies of KIFs have lent credence to the critical role that kinesins play in normal cellular function as well in neuropsychiatric disorders. Mutations of KIFs or their dysfunction are associated with numerous neurological diseases, such as Alzheimer’s disease, Amyotrophic lateral sclerosis, intellectual disability, and microcephaly (Kamal et al., 2001; Makrythanasis et al., 2018; Nicolas et al., 2018). Despite our understanding of the neuronal functions of KIFs, the functions of mitotic KIFs in post-mitotic neurons are less understood. Therefore, we focused on KIF3B, which is known to have a role in both mitotic and post-mitotic neurons. KIF3B exists as a heterotrimer with KIF3A and KAP3, yet it has its own force-generating capacity (Yamazaki et al., 1995). KIF3B also has a critical role in development (Shimizu et al., 1998; Haraguchi et al., 2006). Genetic deletion of KIF3B results in deficits of left-right asymmetry during development in mice (Nonaka et al., 1998). It is believed that KIF3B uses MTs like tracks to traffic cargos required to support protein synthesis (Nonaka et al., 1998; Milic et al., 2017). In the CNS, KIF3B is highly expressed in the retina and is essential for synaptic transmission in photoreceptor cells through axonal transport (Muresan et al., 1999; Marszalek and Goldstein, 2000; Feng et al., 2017). Collectively, these genetic studies suggest that KIF3B has both mitotic and post-mitotic roles. However, the specific role of KIF3B in post-mitotic neurons remains to be described. To address this question, we studied the effect of the post-developmental knockdown of KIF3B in cortical neurons. We used specific shRNAs to knockdown KIF3B in mature cortical neurons and assessed the necessity of KIF3B in modulating neuronal architecture. We found that knockdown of KIF3B resulted in an enhancement in the spine density and the number of thin spines and mushroom spines, in mature neurons. This enhancement was accompanied by a significant increase in the dendritic arbor. By contrast, enhancing levels of KIF3B *via* overexpression decreased dendritic arborization, and spine density. These decreases in dendritic arborization and spine density suggests that KIF3B is a critical component of the mechanisms governing dendritic architecture.

## Materials and Methods

### Animals

Pregnant CD1 mice (Charles River Laboratories) were singly housed on a 12-h light/dark cycle with *ad libitum* access to food and water. All experiments were performed during the light phase of the diurnal cycle. Housing, animal care, and experimental procedures were consistent with the Guide for the Care and Use of Laboratory Animals and approved by the Institutional Animal Care and Use Committee of the Scripps Research Institute.

### Neuronal Cultures, Transfection of shRNAs

Primary cortical cultures were prepared from pooled brains of embryonic day 18 mice of both sexes. Cells were plated at a density of 1 × 10^5^ on poly-D-lysine-coated dishes and glass coverslips. Cultures were plated in Neurobasal medium (Invitrogen) supplemented with 10% fetal bovine serum, while maintained in Neurobasal medium supplemented with 2% B27 (Invitrogen), 0.5 mM GlutaMAX (ThermoFisher), and 1% penicillin/streptomycin solution at 37°C. Neurons were fed every 3–4 days by replacing half of the culture medium with fresh culture media. Mouse, 4 unique 29-mer shRNA constructs against KIF3B in a lentiviral GFP vector (TL501182) with a non-effective 29-mer scrambled shRNA cassette in a pGFP-C-shLenti vector as control (TR30021) were obtained from OriGene. For knockdown experiments, cortical neurons [13–15 days *in vitro* (DIV)] were transfected using Lipofectamine 2000 (Invitrogen) according to the manufacturer’s instructions and allowed to express for 72 h. For KIF3B overexpression, a custom full-length KIF3B-GFP tagged plasmid (EX-Mm33992-Lv103, Genecopoeia) was generated. As a control, pLJM1-EGFP was a gift from David Sabatini (Addgene plasmid #19319^1^; RRID: Addgene_19319; Sancak et al., 2008) and pEGFP-C1 was obtained from Clontech. Neurons were transfected with the plasmid on DIV 10–12 and allowed to express for 48 h using Lipofectamine 2000 (Invitrogen) according to the manufacturer’s instructions. Twenty-four hours before transfection, the neurons were fed and the old media was saved for medium replacement after transfection. Only pyramidal neurons were included in the imaging analysis.

### Lentivirus Package and Concentration

HEK293T cells (CRL-11268, ATCC) were maintained at 37°C and 5% CO_2_ in a humidified atmosphere incubator with a culture media containing DMEM-high glucose (Gibco), 10% fetal bovine serum (ThermoFisher), 1% penicillin-streptomycin (10,000 U/ml, Gibco), and 1% sodium pyruvate (100 mM, Gibco). Twenty-four hours before transfection, HEK293T cells were seeded at a density of 1.9 × 10^7^ per T182 flask.

Lentiviruses were produced by transfecting the HEK293T cells with the shKIF3B and shScrambled vectors expressing GFP and three helper plasmids (pVSVg, pRRE, and pREV; Dull et al., 1998). HEK293T cells were transfected with Lipofectamine 2000 at a ratio of pshKIF3B:pRRE:pREV:pVSVg = 20:13:7:5 (ThermoFisher), following the manufacturer’s instructions. The virus-laden medium was harvested 48 h after transfection and subsequently filtered with a 1,000-rpm centrifuge and a 0.45 μm filtration (Millipore). The filtered medium was then overlaid on a 10% sucrose-PBS cushion and spun at 25,000-rpm at 4°C for 2^1^/_2_ h. After centrifugation, the supernatant was carefully decanted, and the tube was placed on Kimwipes for 5 min to drain. Cold PBS was added to the centrifuge tube and then the tube was placed at 4°C overnight with a parafilm cover for viral recovery.

### Sholl and Spine Analysis

After 48 or 72 h of transfection of cortical neurons with FL-KIF3B or shKIF3B (respectively), images of dendrites were collected at room temperature in the light microscopy facility at the Max Planck Florida Institute, using a confocal microscope (LSM 780; Carl Zeiss; Plan Neofluor 63×/1.3 N.A. Korr differential interference contrast M27 objective in water). Z-stack images were acquired using ZEN 2015 (64 bit) software (Carl Zeiss) and dendritic arbors were manually traced using maximal intensity projection images and later quantified by Sholl analysis FIJI (ImageJ, NIH). The center of the soma was considered the midpoint and the origin of the concentric radii were set from that point to the longest axis of the soma. The parameters set for analysis were: starting radius 20 μm, ending radius 100 μm, radius step size 10 μm. The maximum value of sampled intersections reflecting the highest number of processes/branches in the arbor was calculated and the number of intersections plotted against distance from the soma center in μm. Data were analyzed using one-way ANOVA with Sidak *post hoc* test. Spine morphology was analyzed using MATLAB software developed in the light microscopy facility at the Max Planck Florida Institute. By using a geometric approach, this software automatically detects and quantifies the structure of dendritic spines from the selected secondary branch (100 μm length) in the Z-stack confocal image. The software assigns the detected spines to one of three morphological categories (thin, stubby, or mushroom) based on the difference in structural components of the spines i.e., head, neck, and shaft. Data were analyzed using one-way ANOVA with Tukey *post hoc* test.

### Immunocytochemistry Analysis

Forty-eight or 72 h after transfection of primary cortical neurons with FL-KIF3B or shKIF3B, neurons were processed for immunocytochemistry. The neuronal culture medium was gently removed, and the cells were rinsed once with D-PBS. The cells were fixed with a freshly prepared solution of pre-warmed 4% paraformaldehyde in D-PBS for 15 min. After fixation, the cells were washed with D-PBS for 10 min and then permeabilized in 0.1% Triton X-100 in D-PBS for 20 min. The cells were incubated in 10% normal horse serum in D-PBS with 0.1% Triton X-100 for 1 h to prevent non-specific binding of the primary antibody followed by overnight incubation at 4°C with the primary antibodies: anti-Synaptophysin (1:1,000, ab32594, Abcam), anti-Piccolo (1:1,000, 142104, Synaptic Systems), anti-VGLUT1 (1:1,000, 135-304, Synaptic Systems), anti-PSD-95 (1:1,000, MA1-045, Thermo Fisher Scientific) and anti-GFP (1:4,000, AF4240, Novus Biologicals) in the blocking solution. After overnight incubation, cells were washed thoroughly three times with D-PBS and the immunoreactivity was probed using Alexa 488/546/568/647-conjugated secondary antibodies (1:1,000; Molecular Probes) for 1 h at room temperature. After three washes with D-PBS, the coverslips were mounted using Fluoro-Gel (Electron Microscopy Sciences). Images were acquired by using the Zeiss LSM 880 confocal microscope system with the 63× objective. Fluorescence was measured using ImageJ (NIH). CTCF was calculated using the following formula: Integrated Density of Dendrite − (Area of selected ROI × Mean fluorescence of background readings). All CTCF values were normalized to control.

### Immunoblotting

Neurons were transduced with shScrambled and shKIF3B-LV on DIV 4 and were maintained normally from then on. On DIV 18, the cultures were placed on ice, washed with cold D-PBS, and triturated with Laemmli buffer to lyse the cells. The lysates were briefly boiled and run on a SDS-PAGE gel. The proteins were transferred to a nitrocellulose membrane, then blocked with 5% milk in TBS-T. The membrane was probed overnight with anti-KIF3B (1:1,500, sc-514165, Santa Cruz). After three washes with TBS-T, the membrane was incubated with anti-Mouse-HRP conjugated (1:5,000, 7076S, Cell Signaling). The membrane was washed twice and then developed (SuperSignal West Femto HRP substrate, Thermo Fisher) and imaged (ChemiDoc MP Imaging System, Biorad). Blots were quantified using ImageJ software.

### Quantitative PCR (qPCR)

qPCR was carried out as described previously (Gräff et al., 2014). Quantification of each transcript was normalized to the mouse 18S reference gene following the 2^−ΔΔCt^ method (Livak and Schmittgen, 2001). The student’s *t*-test was used to analyze genes with statistically significant expression levels, where *p-value < 0.05.

### Statistical Analysis

Experiments were performed independently at least three times. “*n*” refers to the number of neurons. All statistical analyses were performed with Prism 7 software (GraphPad). Student’s *t*-test or one-way ANOVA was used for comparison between the groups, and statistical significance was defined as *p* < 0.05. Data used for preparing plots in all figures and corresponding statistical analyses are described in Supplementary Tables 1–8.

## Results

### KIF3B Knockdown Enhances Spine Density and Alters Spine Morphology

**Figure 1:**
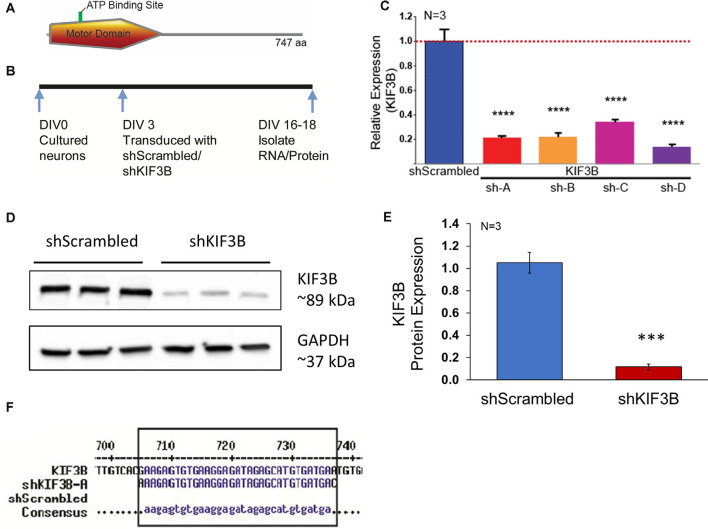
shKIF3B induces a significant reduction in both KIF3B protein and mRNA in cultured cortical neurons. **(A)** Illustration of KIF3B domains. **(B)** Experimental schema. **(C)** Analysis of KIF3B expression from RNA of cortical neurons transduced with shScrambled-LV and shKIF3B-LV, respectively. Ct-values were normalized to 18 s RNA for dCT and ddCT was calculated relative to the scrambled group levels. Error bars are SEM, *N* = 3 per group. **(D)** Western blot of lysates from DIV 18 cortical neurons transduced with shScrambled-LV and shKIF3B-LV, respectively. The blots were probed for KIF3B and GAPDH (loading control). **(E)** The bar graph depicts the densitometry of bands measured using ImageJ (NIH) after normalization to the loading control, ***P < 0.001, ****P < 0.0001. Error bars are SEM. Data used for preparing plots are shown in Supplementary Table 1. **(F)** Sequence alignment was performed with shKIF3B-A and mouse KIF3B mRNA (MultAlin). Data used for preparing plots are shown in Supplementary Table 1.

The mouse, KIF3B ORF, codes for 747 amino acids with the motor domain located at the N-terminal end (Figure 1A). Since genetic studies have suggested that KIF3B functions in cell division and early development, to assess the role of KIF3B in post-mitotic neurons, we considered RNAi mediated loss of function experiments. To carry out the RNAi mediated KIF3B loss of function studies, we obtained four different shRNAs against KIF3B and a non-targeting shRNA control plasmid (shScrambled) with a GFP tag. Primary cortical neurons (DIV 3–4) were transduced with the plasmids to determine their relative knockdown efficiency (Figure 1B). All four plasmids significantly reduced mRNA expression of KIF3B (Figure 1C). Of the four, shKIF3B-A (0.215 ± 0.007) and sh-KIF3B-D (0.140 ± 0.011), showed the greatest knockdown as compared to control, 1.000 ± 0.095 (one-way ANOVA, Tukey *post hoc* test, *F*_(4,10)_ = 60.47, ****p < 0.0001). Based on these results, we used the shKIF3B-A construct along with ShScrambled in subsequent experiments. To further confirm knockdown, we measured the amount of KIF3B protein with shKIF3B-A transduction. As seen with the mRNA, we observed a significant reduction in KIF3B protein when compared to the negative control (Figures 1D,E, Student’s *t*-test, *t*_(5)_ = 7.013, ***p < 0.001). The binding site of this shRNA on KIF3B is shown in Figure 1F.

**Figure 2:**
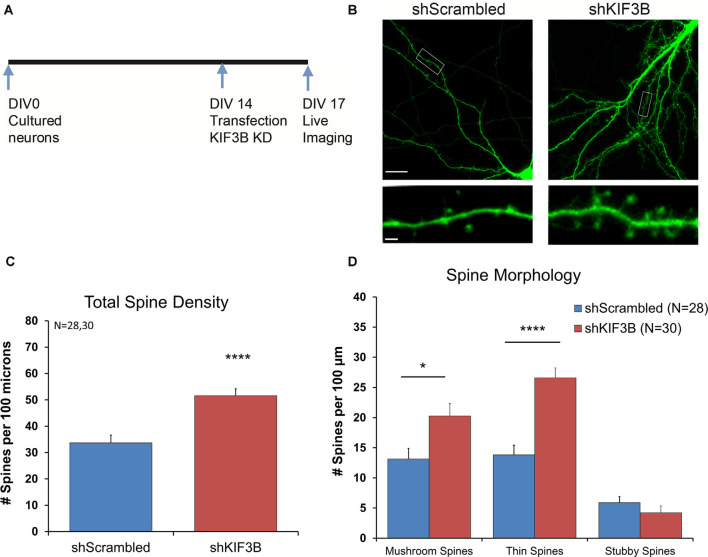
KIF3B knockdown increases overall spine density and morphology in cultured cortical neurons. **(A)** Experimental Schema. **(B)** Representative confocal images of DIV 17 neurons transfected with shScrambled and shKIF3B for 72 h. **(C,D)** Quantification of total spine density and morphology of spines. The analysis was carried out with custom MATLAB software. **(C)** Student *t*-test, ****P < 0.0001. **(D)** Student *t*-test, *P < 0.05, ****P < 0.0001. Scale bar, 20 μm, and 2 μm. N (number of neurons) is indicated per group. Error bars are SEM. Data used for preparing plots are shown in Supplementary Table 2.

We first studied the effect of KIF3B knockdown on spine density and morphology. ShKIF3B-A plasmid was transfected into cortical neurons at DIV 13–14 (Figure 2A) and allowed to express for 72 h following an established protocol in the laboratory (Swarnkar et al., 2018). These cultures were used for live confocal imaging focusing on the dendritic spines of pyramidal neurons since they are the primary sites of input for glutamatergic synapses (Figure 2B). Using a custom-written MATLAB pipeline (Swarnkar et al., 2018), we were able to measure the total spine number per 100 μm as well as determine the morphology of the spines. As depicted in the bar graph (Figure 2C), 72 h of KIF3B knockdown significantly increased the total spine density of pyramidal neurons (shKIF3B 51.56 ± 2.7 and shScrambled 33.69 ± 2.9, Student’s *t*-test, *t*_(56)_ = 4.546, *p* < 0.0001).

Next, we studied whether KIF3B inhibition alters the morphology of dendritic spines. Consistent with the spine density data, we observed an enrichment in the number of mushroom and thin spines; no change in stubby spines was observed (Figure 2D; Mushroom spines: shKIF3B 20.28 ± 2.07, shScrambled 13.14 ± 1.78, Student’s *t*-test, *t*_(56)_ = 2.596, *p < 0.05; Thin spines: shKIF3B-A 26.58 ± 1.65, shScrambled 13.81 ± 1.59, Student’s *t*-test, *t*_(56)_ = 5.546, ****p < 0.0001; Stubby spines: shKIF3B-A 4.23 ± 1.14, shScrambled 5.91 ± 1.00, Student’s *t*-test, *t*_(56)_ = 1.094, ns. *p* > 0.05). These results indicate that KIF3B is a negative regulator of spine density and spine morphology of cortical pyramidal neurons.

### KIF3B Knockdown Increases Dendritic Arborization

**Figure 3:**
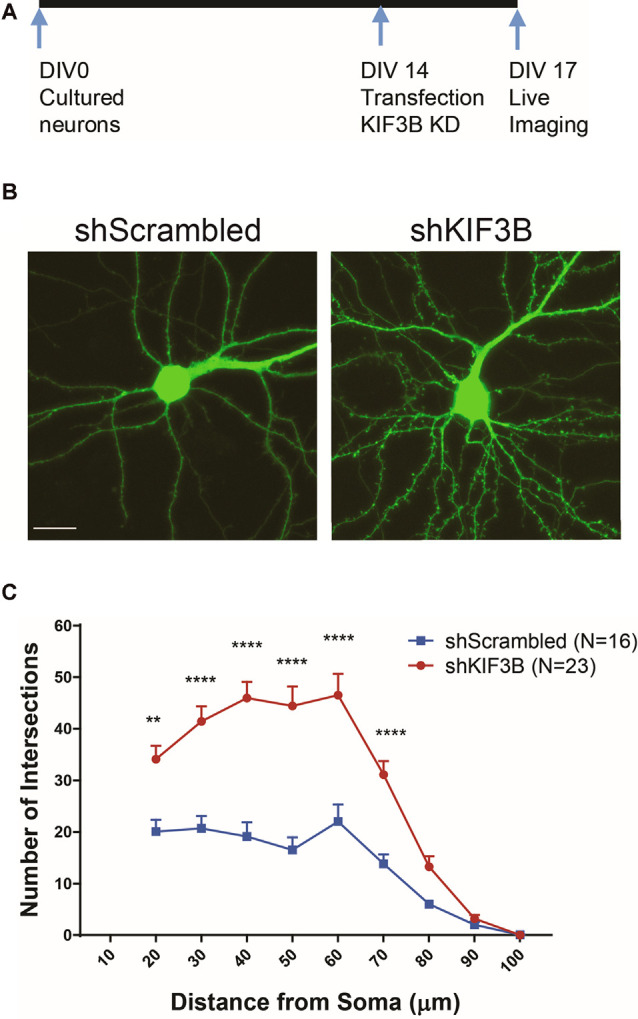
KIF3B knockdown increases dendritic arborization. **(A)** Experimental schema. **(B)** Representative confocal images of cortical neurons transfected with shScrambled and shKIF3B. **(C)** Line graphs depicting the quantification of dendritic arborization (ImageJ, NIH). Two-way repeated-measures ANOVA; **P < 0.01, ****P < 0.0001. Scale bar, 20 μm. N (number of neurons) is indicated per group. Error bars are SEM. Data used for preparing plots are shown in Supplementary Table 3.

Next, we asked whether dendritic arborization could be altered by KIF3B knockdown, consistent with the spine density and morphology data. To address this question, we knocked down KIF3B in cortical cultures at DIV 13–14 for 72 h and imaged the dendritic architecture by confocal imaging (Figures 3A,B). Sholl analysis (Swarnkar et al., 2018) on the resultant confocal images show a significant enhancement in dendritic branching with KIF3B knockdown. From distances of 20 to 70 μm, knockdown of KIF3B dramatically increases the dendritic arborization of cortical pyramidal neurons (Figure 3C, two-way ANOVA with Sidak *post hoc* test, *F*_(1,37)_ = 34.46, ****p < 0.0001). These data suggest that KIF3B acts as an inhibitory constraint of dendritic architecture in cortical neurons.

### KIF3B Preferentially Increases the Distribution of Synaptic Proteins Within the Pre- and Post-synaptic Compartment

Work from other labs has demonstrated that KIF3B participates in fast axonal transport (Yamazaki et al., 1995; Guzik-Lendrum et al., 2015). KIF3B is also known to associate with the presynaptic component in photoreceptor neurons of the retina (Muresan et al., 1998, 1999; Whitehead et al., 1999). Considering this data, we sought to determine whether KIF3B was also involved in the distribution of synaptic proteins within the pre- and post-synaptic compartment. We studied the dendritic distribution of three pre-synaptic proteins (vGLUT1, synaptophysin, piccolo) and one post-synaptic protein (PSD-95) in the presence of KIF3B loss of function. Vesicular glutamate transporter 1, vGLUT1, mediates the reuptake of glutamate into synaptic vesicles (Wojcik et al., 2004) and synaptophysin is a key protein involved in vesicle release (Thomas et al., 1988), while piccolo is a large multimeric protein involved in the formation of the active zone (Mukherjee et al., 2010). PSD-95, on the other hand, is a scaffolding protein that is important for synaptic transmission (Chen et al., 2011, 2015).

**Figure 4:**
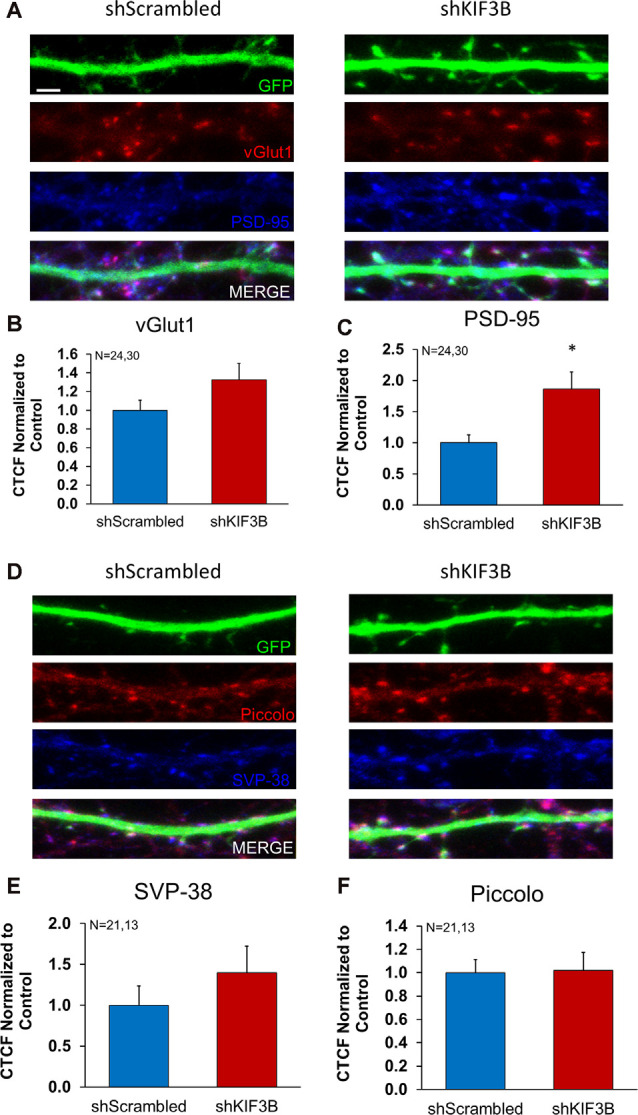
Expression of pre- and postsynaptic proteins in KIF3B knockdown cortical neurons. **(A,D)** Representative confocal images of cortical neurons 72 h after transfection with shScrambled and shKIF3B. **(B,C,E,F)** The bar graph depicts the corrected total cell fluorescence (CTCF) of Synaptophysin, Piccolo, PSD-95, and VGLUT1 analyzed using ImageJ (NIH). Student *t*-test, *P < 0.05. Scale bar, 2 μm, N (number of neurons) is indicated per group. Error bars are SEM. Data used for preparing plots are shown in Supplementary Table 4.

Following the KIF3B knockdown in DIV 16 cortical cultures, GLUT1, synaptophysin, piccolo, and PSD-95 were imaged *via* confocal microscopy (Figures 4A,D). Quantitative analysis of the images (Figures 4B,C,E,F) showed an increase in fluorescence of PSD-95 (shKIF3B 1.865 ± 0.274 as compared to shScrambled 1.000 ± 0.127, Student’s *t*-test, *t*_(52)_ = 2.647, *p < 0.05) with knockdown of KIF3B; though there was no change in vGlut1 (1.324 ± 0.179 as compared to control 1.000 ± 0.109, Student’s *t*-test, *t*_(52)_ = 1.457, ns. *p* > 0.05), piccolo (1.021 ± 0.150 as compared to control 1.000 ± 0.111, Student’s *t*-test, *t*_(32)_ = 0.1139, ns. *p* > 0.05), and synaptophysin (1.399 ± 0.324 as compared to control 1.000 ± 0.236, Student’s *t*-test, *t*_(32)_ = 1.014, *p* > 0.05). Taken together, these results show that KIF3B specifically regulates the distribution of PSD95, a key post-synaptic protein involved in excitatory synaptic transmission.

### Overexpression of KIF3B Produces a Deficit in Spine Density, Morphology, and Dendritic Arborization

Next, we asked whether enhancing the levels of KIF3B might produce a decrease in spine density and alter spine morphology. We assumed that if KIF3B could function as a master regulator of neuronal architecture, we might observe opposite phenotypes with KIF3B knockdown and overexpression. To assess this possibility, we obtained a plasmid that expressed full-length KIF3B (FL-KIF3B) and a GFP tag under the control of the CMV promoter (Supplementary Figure 1A). We assessed the expression levels by KIF3B immune cytochemistry (ICC). Quantitative analysis of immunocytochemical images (Supplementary Figure 1B) showed a 160% increase in KIF3B expression in the dendrites (FL-KIF3B 1.601 ± 0.150 vs. eGFP 1.000 ± 0.072, Student’s *t*-test with Welsh correction, *t*_(15.86)_ = 3.613, **p < 0.01) and a 170% increase in the soma (FL-KIF3B 1.716 ± 0.063 vs. eGFP 1.000 ± 0.069, Student’s *t*-test, *t*_(11)_ = 7.373, ****p < 0.0001, Supplementary Figures 1C,D) of cortical neurons expressing FL-KIF3B when compared to control.

**Figure 5:**
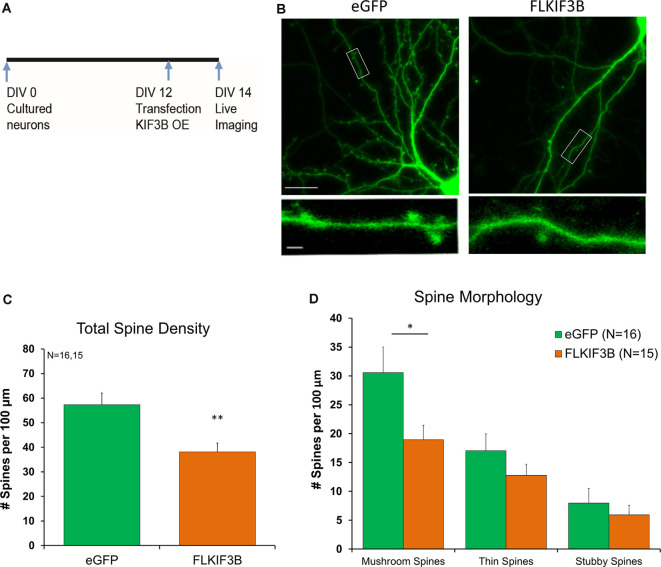
KIF3B overexpression reduces spine density and morphology in cultured cortical neurons. **(A)** Experimental Schema. **(B)** Representative confocal images of DIV 14 neurons transfected with eGFP and FLKIF3B for 48 h. **(C,D)** Quantification of total spine density and morphology of spines. The analysis was carried out with custom MATLAB software. **(C)** Student *t*-test, **P < 0.01. **(D)** Student *t*-test, *P < 0.05. Scale bar, 25 μm, and 5 μm. N (number of neurons) is indicated per group. Error bars are SEM. Data used for preparing plots are shown in Supplementary Table 5.

Similar to the previous protocol, FL-KIF3B was expressed in cortical neurons on DIV 12 (Figure 5A). Two days later, the cultures underwent live-imaging *via* confocal microscopy focusing on dendritic spines (Figure 5B). As depicted in the bar graph (Figure 5C), overexpression of KIF3B, for 48 h, significantly reduces the total spine density of pyramidal neurons (FL-KIF3B 38.13 ± 3.6 and eGFP 57.31 ± 4.8, Student’s *t*-test, *t*_(30)_ = 3.216, **p < 0.001).

Next, we asked whether overexpression of KIF3B had any effect on the morphology of dendritic spines. Complementary to our spine density observations, we saw a reduction in the number of mushroom spines, while thin and stubby spines remained unchanged (Figure 5D; Mushroom spines: FL-KIF3B 18.95 ± 2.50, eGFP 30.55 ± 4.45, Student’s *t*-test, *t*_(30)_ = 2.275, *p < 0.05; Thin spines: FL-KIF3B 12.79 ± 1.94, eGFP 17.08 ± 2.93, Student’s *t-test*, *t*_(30)_ = 1.223, ns. *p* > 0.05; Stubby spines: FL-KIF3B 5.93 ± 1.68, eGFP 7.98 ± 2.52, Student’s *t-test*, *t*_(30)_ = 0.6782, ns. *p* > 0.05). These results are consistent with KIF3B being a negative regulator of spine density and spine morphology of cortical pyramidal neurons.

**Figure 6:**
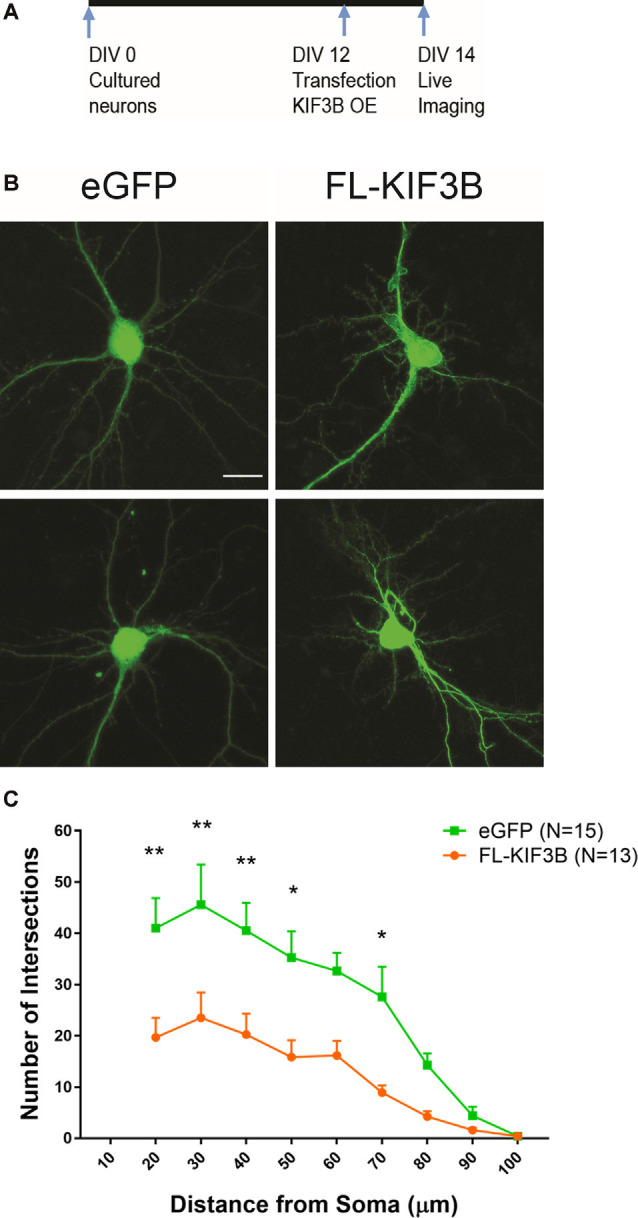
KIF3B overexpression reduces dendritic arborization. **(A)** Experimental schema. **(B)** Representative confocal images of cortical neurons transfected with eGFP and FLKIF3B. **(C)** Line graphs depicting the quantification of dendritic arborization (ImageJ, NIH). Two-way ANOVA, **P < 0.01 with Sidak *post hoc* test, *P < 0.05, **P < 0.01. Scale bar, 20 μm. N (number of neurons) is indicated per group. Error bars are SEM. Data used for preparing plots are shown in Supplementary Table 6.

Next, we studied the effect of overexpression of KIF3B in dendritic arborization. Cortical neurons at DIV 12 were transfected with KIF3B, full-length expressing plasmid (Figure 6A). Forty-eight hours later, the dendrites of the neurons were live-imaged *via* confocal microscopy (Figure 6B). Sholl analysis showed a significant reduction in dendritic branching with overexpression of KIF3B. Over the distance of 20 to 70 μm, the expression of FL-KIF3B reduced the dendritic arborization of cortical pyramidal neurons (Figure 6C; two-way ANOVA with Sidak *post hoc* test, *F*_(1,25)_ = 12.91, **p < 0.01). Taken collectively, these data further suggest that KIF3B functions as a negative regulator of the dendritic architecture of mature neurons.

**Figure 7:**
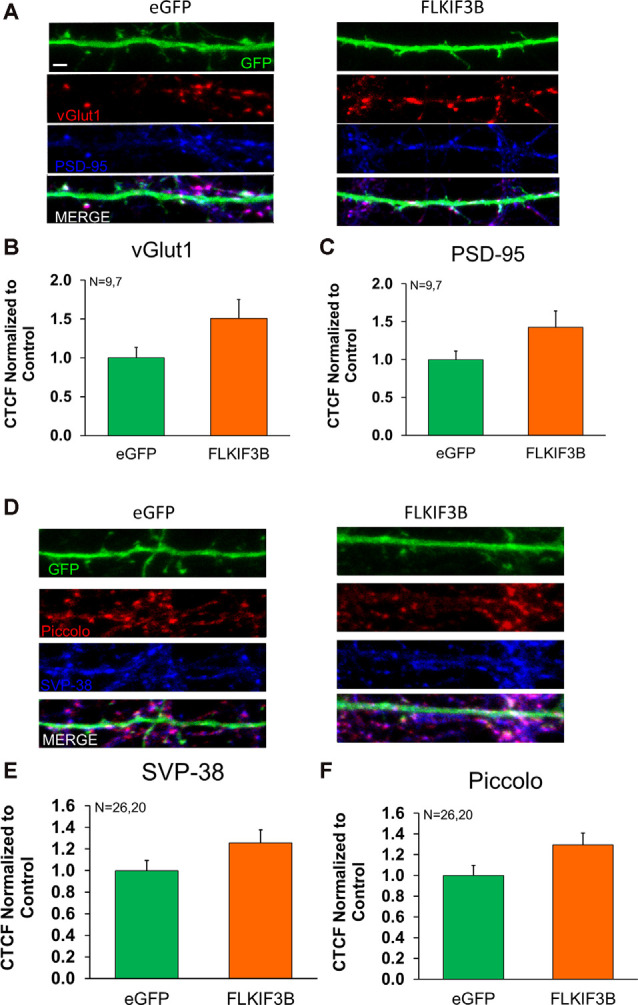
Expression of pre- and postsynaptic proteins in KIF3B overexpressed cortical neurons. **(A,D)** Representative confocal images of cortical neurons 48 h after transfection with eGFP and FLKIF3B. **(B,C,E,F)** The bar graph depicts the total number of PSD-95 and VGLUT1 punta per μm of GFP. The analysis was performed using ImageJ (NIH). Student *t*-test, ns *P* > 0.05. Scale bar, 2 μm, N (number of neurons) is indicated per group. Error bars are SEM. Data used for preparing plots are shown in Supplementary Table 7.

Last, we sought to know whether overexpression of KIF3B had any effect on the distribution of proteins within the pre- and post-synaptic compartments. To accomplish this, we overexpressed KIF3B in cortical neuronal cultures at DIV 12 and 48 h later performed immunocytochemistry against vGLUT1, synaptophysin, piccolo, and PSD-95 (Figures 7A,D). Quantitative analysis of the images (Figures 7B,C,E,F) showed no change in fluorescence of PSD-95 with overexpression of KIF3B (FLKIF3B 1.424 ± 0.217 as compared to eGFP 1.000 ± 0.112, Student’s *t*-test, *t*_(14)_ = 1.94 ns. *p* > 0.05), vGLUT1 (1.509 ± 0.244 as compared to control 1.000 ± 0.134, Student’s *t*-test, *t*_(14)_ = 1.856 ns. *p* > 0.05), piccolo (1.297 ± 0.113 as compared to control 1.000 ± 0.097, Student’s *t*-test, *t*_(44)_ = 1.993, ns. *p* > 0.05), and synaptophysin (1.258 ± 0.119 as compared to control 1.000 ± 0.093, Student’s *t*-test, *t*_(44)_ = 1.735, ns. *p* > 0.05). Taken together, these results show that overexpression of KIF3B does not alter the distribution of PSD95.

## Discussion

The significance of KIFs in cell division, synaptic plasticity, and memory has previously been described (Miki et al., 2005; Hirokawa et al., 2009). KIFs are mostly known for their ability to mediate transport of gene products such as proteins, RNAs, and organelles required for neuronal functioning in diverse organisms such as sea slug (*Aplysia californica*), fruit fly (*Drosophila melanogaster*), worm (*Caenorhabditis elegans*), and mammals (*Mus musculus*). Several synaptic proteins such as piccolo, bassoon, GRIP, PSD95, and RNAs such as myosin heavy chain, CaMK kinase II are associated with specific KIFs. Furthermore, KIFs mediate the transport of organelles such as synaptic vesicles and mitochondria (Sato-Yoshitake et al., 1992; Okada et al., 1995; Pilling et al., 2006). These studies demonstrate that KIFs are a major regulator of neuronal development and function.

Apart from their transport role, KIFs play a vital role in cell division. Several KIFs are known to be involved in spindle assembly and movement (Hirokawa, 2000). Functions of these mitotic KIFs include generating forces during spindle movement as well as transporting specific proteins. However, recent studies have suggested that some of these mitotic KIFs also function in post-mitotic neurons. We have previously shown that mitotic KIFs, such as KIF11 function in post-mitotic neurons by imposing inhibitory constraints on synaptic transmission (Swarnkar et al., 2018). Importantly, when KIF11 was knocked down, we observed an increase in synaptic transmission. Furthermore, the KIF11 knockdown resulted in an increase in dendritic arborization as well as the number of mushrooms and thin spines.

Due to the lack of known KIFs that pose inhibitory constraints in post-mitotic neurons, we searched for additional KIFs with similar properties. We particularly focused on KIFs that were critical for cell division but were also associated with transporting cargos in post-mitotic neurons. KIF3B fit these criteria and we assessed the effect of KIF3B knockdown on neuronal architecture. KIF3B functions as a heterotrimer, forming a complex with KIF3A and KAP3. Importantly, we know little about KIF3B’s function in mature neurons.

Our analysis of post-mitotic KIF3B knockdown showed enhanced spine density, an increase in the number of thin and mushroom spines, and an increase in dendritic arborization, wholly suggesting global neuronal changes. These enhancements in key features of neuronal architecture suggest that KIF3B functions as an inhibitory constraint on structural plasticity. It is possible that KIF3B, or one of its cargos, inhibits microtubule elongation. Once KIF3B inhibition is relieved, microtubule elongation is enhanced, facilitating dendritic arborization and synapse density. Indeed, changes in dendritic arborization are supportive of the enhanced microtubule growth in KIF3B knockdown neurons. Thus, regulation of KIF3B function would be an intriguing mechanism in which to check microtubule elongation in mature neurons.

Next, we searched for synaptic proteins whose expression is altered in KIF3B knockdown neurons. We examined the localization of three key presynaptic proteins (Piccolo, Synaptophysin, and vGLUT1) and a postsynaptic protein (PSD95). Piccolo is an active zone protein and synaptophysin is a synaptic vesicle protein. Both are critical for synaptic transmission (Thomas et al., 1988; Mukherjee et al., 2010). Both vGLUT1 and PSD95 are markers for excitatory synapses (Thomas et al., 1988; Chen et al., 2015). We found specific changes in the expression of these proteins in KIF3B knockdown neurons. Specifically, vGLUT1, piccolo, and synaptophysin did not change in their expression, whereas there was a significant enhancement in PSD95 immunoreactivity in KIF3B knockdown neurons. These results indicate that KIF3B may have a postsynaptic effect in excitatory neurons. Further, the increase of PSD-95 at the synapse may indicate the presence of mature spines. This assertion is bolstered by the enhancement in the number of mushroom spines observed with KIF3B knockdown. Interestingly, KIF3B overexpression did not affect the distribution of PSD95 and vGLUT1. Considering the global decrease in dendritic and synaptic architecture with KIF3B overexpression, these results suggest that KIF3B primarily alters dendritic arborization, which eventually results in the altered distribution of synaptic proteins, spine density, and morphology.

The increase in PSD-95 immunoreactivity is particularly interesting when we compare the effects of knockdown of KIF11, another KIF that we previously reported to act as an inhibitory constraint. KIF11 knockdown neurons showed enhanced piccolo and synaptophysin expression whereas expression of PSD95 was not altered (Swarnkar et al., 2018). These results suggest there are unique ways by which KIFs function as inhibitory constraints on structural plasticity.

Recent work has shown that KIF3B may be involved in the trafficking of the NMDAR subunit, NR2A (Alsabban et al., 2020). In this work, KIF3B knockdown showed a contrasting phenotype, namely a reduction in spine density, stubby spines, and PSD-95 expression. There are two possible reasons for this discrepancy. The authors use a germline mutant mouse to reduce the levels of KIF3B. In this scenario, the contrasting phenotype is more indicative of a developmental loss of KIF3B. Second, many of the experiments were carried out in hippocampal neurons. Work from our lab has demonstrated that though hippocampal and cortical neurons may express the same KIFs, their cargos may differ, hence, function differently in specific tissues (Liu et al., 2014). Thus, restricting KIF3B knockdown to mature neurons allows the study of KIF3B function apart from development.

In summary, our results suggest that KIF3B acts as a negative regulator of dendritic architecture. Understanding the detailed mechanisms by which KIFs that function as inhibitory constraints in post-mitotic neurons is expected to shed novel insights into the regulation of KIFs in modulating neuronal architecture and synapse function.

## Data Availability Statement

All datasets generated for this study are included in the article.

## Ethics Statement

The animal study was reviewed and approved by the Scripps IACUC Commitee.

## Author Contributions

NJ and SP designed the research, generated the first draft and revised it with inputs from all authors. NJ did imaging and morphology, gene expression analyses, immunocytochemistry, immunoblotting, generation of reagents for KIF manipulations, and statistical analyses of data. EG performed imaging and morphology of blind replication study. SS transfected neurons for the blind replication study. All authors contributed to the article and approved the submitted version.

## Conflict of Interest

The authors declare that the research was conducted in the absence of any commercial or financial relationships that could be construed as a potential conflict of interest.

## Footnotes

^1^http://n2t.net/addgene:19319

## Supplementary Material

SUPPLEMENTARY FIGURE 1 Characterization of KIF3B overexpression. **(A)** Map of FL-KIF3B ORF plasmid. **(B)** Representative confocal images of cortical neurons transfected with eGFP and FL-KIF3B. **(C,D)** Bar graph depicts the corrected total cell fluorescence (CTCF) of KIF3B expression in the dendrites and soma, analyzed using ImageJ (NIH). Student *t*-test ***P* < 0.01, *****P* < 0.0001. Scale bar, 25 μm, and 5 μm. N is indicated per group. Error bars are SEM. Data used for preparing plots are shown in Supplementary Table 8.
