# Supplementary figures and images for: Molecular Motor KIF3B Acts as a Key Regulator of Dendritic Architecture in Cortical Neurons
Source: Front Cell Neurosci. 2020 Oct 19;14:521199. doi: 10.3389/fncel.2020.521199 (PMC7604319; doi:10.3389/fncel.2020.521199)

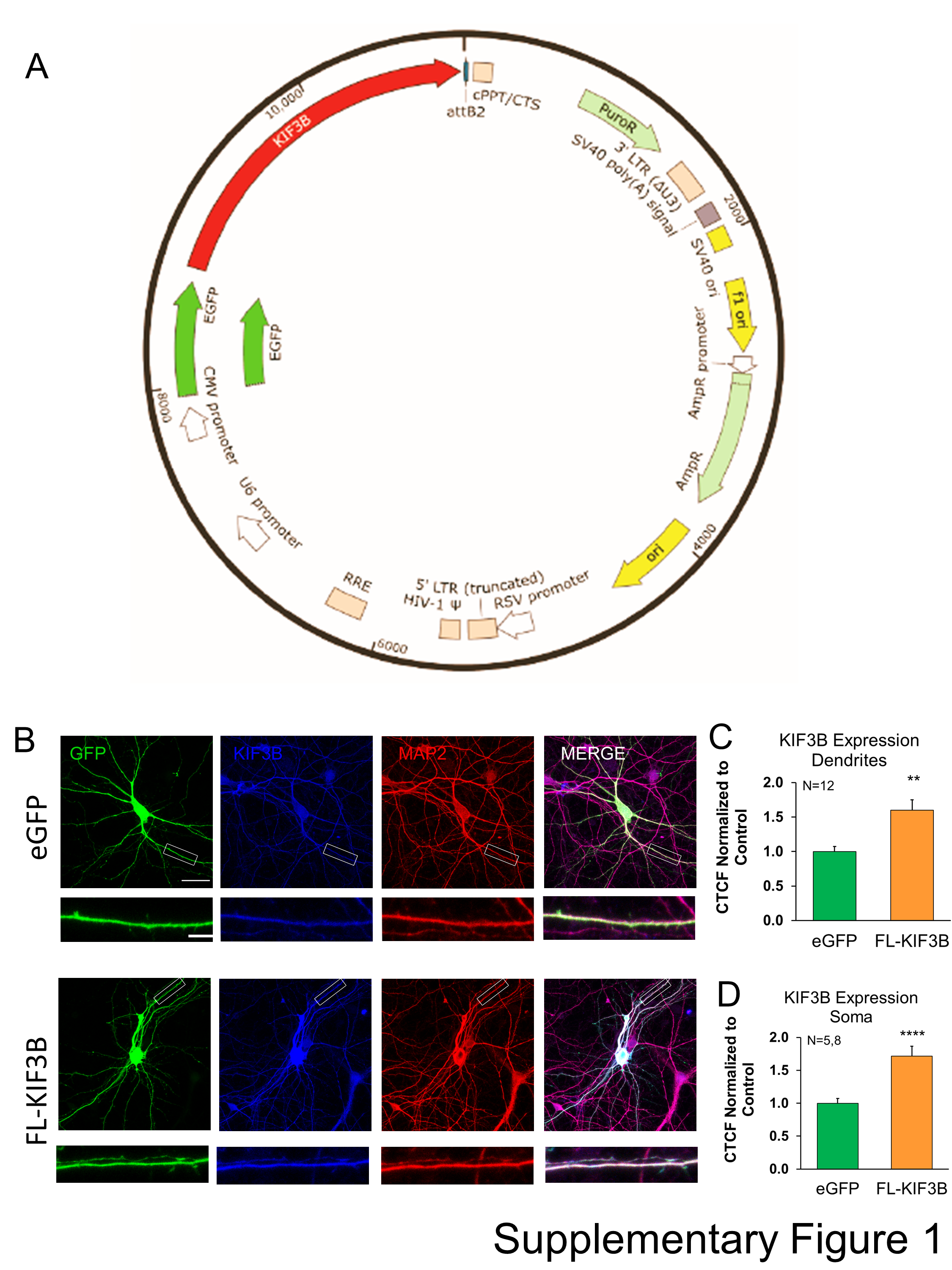

Supplement: SUPPLEMENTARY FIGURE 1 — Characterization of KIF3B overexpression. (A) Map of FL-KIF3B ORF plasmid. (B) Representative confocal images of cortical neurons transfected with eGFP and FL-KIF3B. (C,D) Bar graph depicts the corrected total cell fluorescence (CTCF) of KIF3B expression in the dendrites and soma, analyzed using ImageJ (NIH). Student t-test **P < 0.01, ****P < 0.0001. Scale bar, 25 μm, and 5 μm. N is indicated per group. Error bars are SEM. Data used for preparing plots are shown in Supplementary Table 8. [file Image_1.tif]
